# Risk factors and predictive value of lung ultrasound aeration score for postoperative pulmonary complications in pediatric patients undergoing abdominal tumor resection: a prospective observational study

**DOI:** 10.1186/s12871-026-04265-1

**Published:** 2026-09-19

**Authors:** Jie Guo, Li-Quan Huang, Zhi-Xiang Yu, Ting-Xuan Yan, Shou-Cai Xu, Jing-Fu Wang, Tao Jiang, Guang-Fen Zhang, Ping Zhao

**Affiliations:** 1 Department of Anesthesiology, Shandong Cancer Hospital and Institute, Shandong First Medical University, Shandong Academy of Medical Sciences, Jinan, 250117 P. R. China; 2 Department of Pediatric Oncology, Shandong Cancer Hospital and Institute, Shandong First Medical University and Shandong Academy of Medical Sciences, Jinan, 250117 P. R. China; 3 Department of Anesthesiology, Shandong Provincial Hospital Affiliated to Shandong First Medical University, Jinan, 250021 P. R. China; 4 Department of Anesthesiology, Shengjing Hospital of China Medical University, Shenyang, 110004 P. R. China

**Keywords:** Pediatric patients, Abdominal malignant tumor resection, Postoperative Pulmonary Complications (PPCs), Risk factors, Lung ultrasound

## Abstract

**Background:**

Postoperative pulmonary complications (PPCs) constitute a leading source of perioperative morbidity in children undergoing abdominal malignant tumor resection. However, the risk factors of PPCs in this specific pediatric population remains unclear, and effective predictive tools are lacking. This study aimed to identify the risk factors for PPCs and evaluate the predictive value of the post-anesthesia care unit (PACU) lung ultrasound aeration score (LUSS).

**Methods:**

This single-center prospective observational study was conducted at a tertiary cancer center in China. Pediatric patients aged 1–18 years scheduled for abdominal malignant tumor resection were enrolled between January 2025 and January 2026. Perioperative clinical data and PACU LUSS measurements were collected. Univariate and multivariate logistic regression analyses were performed to identify independent risk factors for PPCs. Receiver operating characteristic (ROC) curve analysis, calibration analysis, and decision curve analysis (DCA) were adopted to quantify the predictive performance of relevant indicators.

**Results:**

The overall incidence of PPCs was 60.98% (150/246). Multivariate logistic regression analysis confirmed that American Society of Anesthesiologists (ASA) classification (OR = 3.60, 95% CI = 1.77–7.34, *P* < 0.001), recent resolved upper respiratory tract infection (URI) (OR = 4.71, 95% CI = 1.84–12.08, *P* = 0.001), duration of mechanical ventilation (OR = 1.01, 95% CI = 1.01–1.01, *P* = 0.002), and PACU LUSS (OR = 1.29, 95% CI = 1.17–1.42, *P* < 0.001) were independent risk factors for PPCs. ROC curve analysis revealed that PACU LUSS alone exhibited favorable predictive efficacy for PPCs, with an area under the ROC curve (AUC) of 0.80. A combined predictive model integrating LUSS and the other three independent risk factors yielded an elevated AUC of 0.88. Calibration curves demonstrated strong consistency between predicted and actual PPCs risks, and DCA verified satisfactory clinical utility and net clinical benefit of the combined model.

**Conclusions:**

PPCs are highly prevalent in pediatric patients undergoing abdominal malignant tumor resection.

Higher ASA classification, recent resolved URI, prolonged duration of mechanical ventilation, and elevated PACU LUSS may be associated with an increased risk of PPCs. As a non-invasive bedside assessment tool, PACU LUSS may provide useful predictive information and could potentially assist with perioperative risk stratification and targeted prevention of PPCs in this vulnerable pediatric population.

**Supplementary Information:**

The online version contains supplementary material available at https://doi.org/10.1186/s12871-026-04265-1.

## Introduction

PPCs are prevalent and clinically impactful adverse events in pediatric surgical practice [1, 2]. They are particularly prevalent in children undergoing abdominal malignant tumor resection, a procedure that serves as an irreplaceable core component of multimodal anti-tumor therapy [3, 4].Preoperative chemotherapy, surgical trauma, and general anesthesia collectively induce physiological disturbances, which markedly elevate the risk of PPCs, prolonging hospital stays, and imposing substantial clinical and familial burdens on pediatric patients [5, 6].

Numerous prior studies have identified PPCs risk factors [7, 8] and constructed predictive models [9, 10] for PPCs adult surgical populations, yet these findings cannot be directly extrapolated to the pediatric population. Pediatric patients feature immature respiratory physiology, characterized by narrower airways and higher basal oxygen consumption [11]. Furthermore, evidence derived from other pediatric surgical disciplines lacks generalizability to abdominal tumor resection due to marked discrepancies in surgical trauma magnitude and physiological stress response [12]. Currently, research on PPCs after pediatric abdominal tumor resection remains limited, with unclear risk factors, making early risk stratification clinically valuable.

Lung ultrasound (LUS) represents a safe, real-time, repeatable bedside imaging modality [13–16].Validated lung aeration scoring systems based on LUS have been proven capable of predicting PPCs in adult cohorts [17–19]. LUS offers clear advantages over X-ray and computed tomography (CT) in children due to favorable acoustic windows [20, 21]. The PACU period represents a critical window for perioperative respiratory monitoring [16, 22]. Lung aeration assessed by LUS in PACU may predict subsequent PPCs, yet no prospective cohort study has validated this in children undergoing abdominal tumor resection.

Accordingly, a single-center prospective observational study was conducted at a tertiary cancer hospital in China, enrolling children aged 1–18 years undergoing abdominal malignant tumor resection. This study had three primary objectives: (1) to quantify the overall incidence of PPCs in this patient population; (2) to identify independent perioperative risk factors for PPCs; (3) to evaluate the predictive value of PACU LUSS for PPCs development. The findings may help identify high-risk children early and support the standardized perioperative use of LUS.

## Methods

### Ethical approval

This study was approved by the Ethics Committee of the Affiliated Cancer Hospital of Shandong First Medical University (Approval number: SDTHEC202412011) and registered in the Chinese Clinical Trials Registry (ChiCTR2500095469). Written informed consent was obtained from the guardians of all pediatric participants prior to study initiation.

### Study design

A single-center prospective observational study was conducted at the Affiliated Cancer Hospital of Shandong First Medical University from January 8, 2025, to January 30, 2026. This study was reported in accordance with the STROBE (Strengthening the Reporting of Observational Studies in Epidemiology) statement.

### Population

Inclusion criteria: (1) Aged 1–18 years; (2) ASA physical status classification I–III; (3) Elective surgical resection of pediatric abdominal tumors, including retroperitoneal neuroblastoma, hepatoblastoma, nephroblastoma, and rhabdomyosarcoma.

Exclusion criteria: (1) Concurrent thoracoscopic or thoracotomy surgery; (2) Emergency surgery; (3) Children whose family members refused participation; (4) Patients who voluntarily withdrew midway; (5) Children requiring reoperation due to postoperative complications within the 7 day postoperative follow up period; (6) Children who had undergone endotracheal intubation or tracheotomy before surgery; (7) Children requiring transfer to the Pediatric Intensive Care Unit for monitoring and treatment after surgery.

### Anesthesia protocol and ventilator settings

Preoperatively, pediatricians and anesthesiologists conduct a comprehensive evaluation of multiple organ systems in pediatric patients. Specialized evaluation was prioritized for patients with a recent URI history, incorporating physical examination, complete blood count, C-reactive protein, procalcitonin, and chest radiography or CT scanning. Elective surgery was rescheduled only after all laboratory markers normalized and URI clinical symptoms resolved completely for a minimum of two weeks.

Standardized preoperative fasting protocols were implemented: 8 h for high-fat solid food, 6 h for starchy solids and milk powder, 4 h for breast milk, and 2 h for clear water. In the pre-induction room, parents were permitted to accompany the children. Following sedation with esketamine 0.3 mg·kg⁻¹ and propofol 1 mg·kg⁻¹, the children were transferred to the operating room for standardized intravenous anesthesia induction. Intraoperatively, continuous monitoring of electrocardiogram, blood pressure, heart rate, and peripheral oxygen saturation was performed.

Anesthesia induction was achieved with an intravenous drug combination: midazolam 0.2 mg·kg⁻¹, sufentanil 0.3–0.5 µg·kg⁻¹, propofol 2 mg·kg⁻¹, and cisatracurium 0.15 mg·kg⁻¹. Anesthesia maintenance was performed with a combination of continuous intravenous infusions (propofol 2 mg·kg⁻¹·h⁻¹, remifentanil 0.1–0.2 µg·kg⁻¹·h⁻¹, cisatracurium 0.1 mg·kg⁻¹·h⁻¹) and inhaled sevoflurane at 1–2 vol%. Anesthetic depth was titrated according to bispectral index monitoring, targeting a range of 40–60. Crystalloid fluid was infused at a rate of 5–10 mL·kg⁻¹·h⁻¹.

Intraoperative volume-controlled mechanical ventilation was delivered via a Draeger ventilator (A350XL), tidal volume 6–8 mL·kg⁻¹ (based on predicted body weight), fresh gas flow 2 L·min⁻¹, fraction of inspired oxygen (FiO₂) 0.4–0.6, inspiratory-to-expiratory ratio 1:2, respiratory rate adjusted to maintain end-tidal carbon dioxide (ETCO₂) 35–45 mmHg, and positive end-expiratory pressure (PEEP) 0–5 cm H₂O, maintained until surgery ends. Zero end-expiratory pressure was primarily used in patients with good respiratory function undergoing hepatectomy to reduce intrathoracic pressure and central venous pressure, thereby optimizing hemodynamics. In contrast, patients undergoing non-hepatic surgery received 3–5 cm H₂O PEEP to maintain alveolar recruitment and intraoperative respiratory stability.

At surgical completion, neuromuscular blockade was reversed with neostigmine 0.04 mg·kg⁻¹ combined with atropine 0.02 mg·kg⁻¹. After completion of surgery, the endotracheal tube was removed once the child resumed adequate spontaneous breathing (tidal volume 6–8 mL·kg⁻¹, ETCO₂ 35–45 mmHg) following thorough oropharyngeal suctioning.Postoperative pain control utilized patient-controlled intravenous analgesia: sufentanil 3 µg·kg⁻¹ diluted in 100 mL normal saline, with a 2 mL·h⁻¹ background infusion, 0.5 mL bolus dose, and a 15-minute lockout interval.

### Definition and assessment of postoperative pulmonary complications

PPCs were defined as new-onset respiratory adverse events occurring within seven days after surgery, including respiratory infection, respiratory failure, pleural effusion, atelectasis, pneumothorax, bronchospasm, and aspiration pneumonitis [9, 23, 24]. For patients discharged within 7 days, follow-up was terminated on the day of discharge.

The definition of respiratory infection required the presence of the following criteria: when a patient received antibiotics for a suspected respiratory infection and met at least one of the following criteria, new or altered sputum properties, new or progressive pulmonary opacities on imaging, fever, and leukocytosis with a white blood cell count exceeding 12 000 µL⁻¹ [9]. The criteria for respiratory failure were defined as follows: after surgery, under room air conditions, arterial partial pressure of oxygen (PaO₂) < 60 mmHg; or the ratio of arterial partial pressure of oxygen to fraction of inspired oxygen (PaO₂/FiO₂) < 300; or arterial oxygen saturation measured by pulse oximetry (SpO₂) < 90% requiring oxygen therapy [9]. Pleural effusion, atelectasis, and pneumothorax were defined based on the findings of chest X-ray, CT, or LUS [9, 25–28].

To minimize subjective bias in lung ultrasound evaluation, all examinations were performed using a standardized protocol, and images were archived for offline analysis. Two physicians independently interpreted the images, and atelectasis or pleural effusion was defined as new or worsened abnormalities compared with baseline, in combination with clinical manifestations. To avoid overinterpretation of minor findings, only lesions requiring clinical intervention including oxygen therapy, antibiotic administration, correction of hypoproteinemia, respiratory physiotherapy, and thoracentesis were regarded as clinically significant. The severity of postoperative pulmonary complications during hospitalization was assessed using an I–V ordinal scale, based on the Clavien-Dindo Classification [29]. Grade I: Any deviation from the normal postoperative course without the need for pharmacological treatment or surgical, endoscopic, and radiological interventions; Grade IV: A life-threatening complication; Grade V: In-hospital death before discharge.

### Data collection

Perioperative data were categorized into preoperative baseline, intraoperative, and postoperative variables as detailed below:

#### Preoperative baseline variables

Age, sex, body mass index (BMI), ASA classification, and recent URI (defined as the presence of URI within 2 to 4 weeks before the actual date of surgery, including both newly developed infections and cases with ongoing symptoms during this period), tumor three-dimensional diameters (tumor volume calculated via the ellipsoid formula $\mathrm{V}=\uppi/6\times\mathrm{length}\times\mathrm{width}\times\mathrm{height}$ [30]), routine laboratory indicators (white blood cell count, neutrophil count, neutrophil percentage, hemoglobin, procalcitonin, C-reactive protein, N-terminal pro-B-type natriuretic peptide, albumin, prealbumin, creatinine, blood urea nitrogen), anesthesiologist seniority, pathological tumor diagnosis, preoperative chemotherapy history, multiple/repeat surgical history, preoperative blood transfusion, and preoperative hypoalbuminemia.

#### Intraoperative variables

Mechanical ventilation duration, total operative time, PACU length of stay, total fluid and blood transfusion volume, intraoperative blood loss, urine output, and key ventilation parameters.

#### Postoperative Variables

Postoperative variables comprised PACU lung ultrasound scores, incidence of PPCs, and length of hospital stay.

### LUS examination

All children underwent lung ultrasound examination using the same Mindray portable color Doppler ultrasound system (M9 Super). A 4–12 MHz linear array transducer was used in most subjects, whereas a 1–5 MHz convex array transducer was employed for older children or those with a thick chest wall. All bedside lung ultrasound examinations were performed via intercostal longitudinal scanning in the PACU 20 min after extubation, conducted by a physician with at least 100 cases of experience in pediatric lung ultrasound in accordance with international guidelines.

**Fig. 1:**
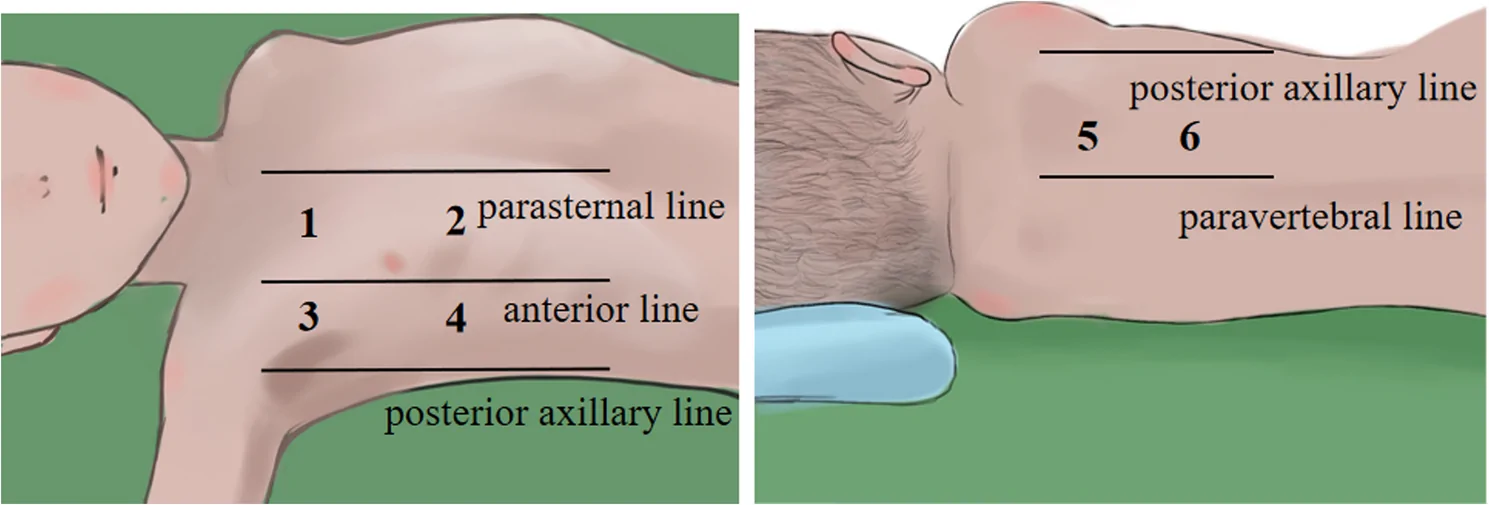
Zones followed for lung ultrasound examination

**Fig. 2:**
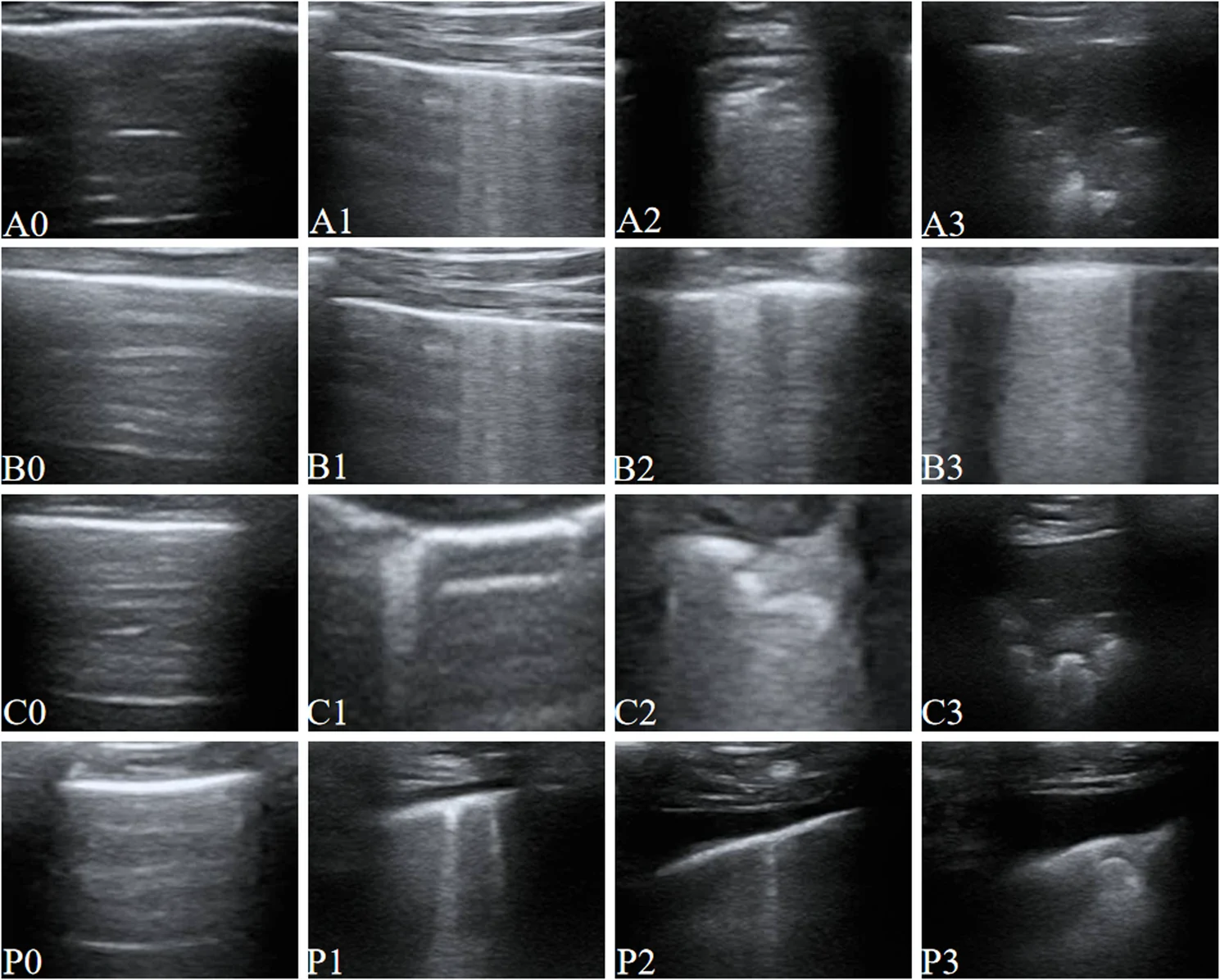
Example of lung ultrasound score. The LUSS was classified into four grades: A0: normal ventilation: A-lines visible, normal pleural sliding, no more than 2 scattered B-lines present, and no lung consolidation; A1: mild ventilation loss: ≥3 scattered B-lines visible (without white lung manifestation), partial A-lines still observable, accompanied by tiny juxtapleural consolidation (diameter < 0.5 cm); A2: moderate ventilation loss: coalescent B-lines visible (forming a white lung sign with no visible A-lines), or the presence of juxtapleural consolidation (consolidation thickness ≤ 1 cm in children, ≤ 0.5 cm/kg in neonates); A3: severe ventilation loss: extensive tissue-like consolidation visible (consolidation depth > 1 cm in children; > 1 cm or > 0.5 cm·kg⁻¹ in neonates).The degree of B-lines lung ultrasound score was divided into four grades: B0: fewer than three isolated B-lines; B1: multiple well-defined B-lines; B2: multiple coalescent B-lines; B3: white lung. The degree of consolidation lung ultrasound score was divided into four grades: C0: no consolidation; C1: minimal juxtapleural consolidation; C2: small-sized consolidation; C3: large-sized consolidation. The degree of pleural effusion lung ultrasound score was divided into four grades: P0: no pleural effusion; P1: minimal pleural effusion; P2: a small amount of pleural effusion; P3: a moderate amount of pleural effusion

Each hemithorax was divided into six anatomical regions: anterior-superior, anterior-inferior, lateral-superior, lateral-inferior, posterior-superior, and posterior-inferior, resulting in a total of 12 regions bilaterally (Fig. 1). Each region was scored using a 0–3 scale, and the sum of scores across all regions yielded the total lung ultrasound score, ranging from 0 to 36. The LUSS was comprehensively determined based on the number and confluence of B-lines, extent of lung consolidation, volume of pleural effusion, and severity of impaired pulmonary aeration [31]. In addition to calculating the LUSS, this study simultaneously evaluated multiple individual dimensional parameters on lung ultrasound, including B-line score, lung consolidation score, and pleural effusion score, using the grading system reported by Song et al. [15] (Fig. 2).

### Statistical analysis

The sample size was calculated using the events per variable (EPV) method for logistic regression. Based on the preliminary trial results, the incidence of PPCs in pediatric patients with abdominal tumors was 64.2%. Ten variables were included in the multivariate regression analysis, including age, operation time, anesthesia duration, ASA classification, hypoproteinemia, intraoperative blood transfusion, preoperative anemia, preoperative chemotherapy, recent upper respiratory tract infection, and reoperation or multiple operations. The EPV was set at 15, and considering a 5% loss to follow-up rate, the calculated required sample size for this study was no less than 10 × 15 ÷ 0.642 ÷ 0.95 = 246 cases. Post-hoc power analysis was performed to verify the adequacy of the sample size. After stepwise screening using univariate regression and collinearity assessment, a total of 15 predictors were finally included in the analysis. A total of 150 cases of PPCs were observed in our study cohort, yielding an EPV ratio of 10, which satisfies the widely accepted statistical criterion of EPV ≥ 10 for logistic regression analysis.

All statistical analyses were performed using R software (version 4.3.0), with core packages including pROC, rms, and rmda for predictive modeling and DCA. Normally distributed continuous variables were expressed as mean ± standard deviation and compared via independent-samples t-tests. Non-normally distributed continuous variables were reported as median (interquartile range) [M (Q1, Q3)] and analyzed using the Mann–Whitney U test. Categorical variables were compared using the chi-square test.

Variables were selected as follows. First, baseline variables with statistically significant intergroup differences (*P* < 0.05) or clinically important value were included in univariate regression analysis to construct an initial candidate variable set. Collinearity diagnosis was then performed for all candidate indicators; variables with a variance inflation factor (VIF) > 5 were eliminated, and clinically representative core indicators were retained while redundant sub-items with low explanatory power were excluded to reduce collinearity bias. Subsequently, least absolute shrinkage and selection operator (LASSO) regression was applied for further dimensionality reduction and screening of key predictors were entered into multivariate logistic regression to identify independent risk factors for PPCs. The predictive performance of the risk factors was assessed using ROC curve analysis, calibration analysis, and decision curve analysis. P value < 0.05 was considered statistically significant.

## Results

### Baseline characteristics of the study population

**Fig. 3:**
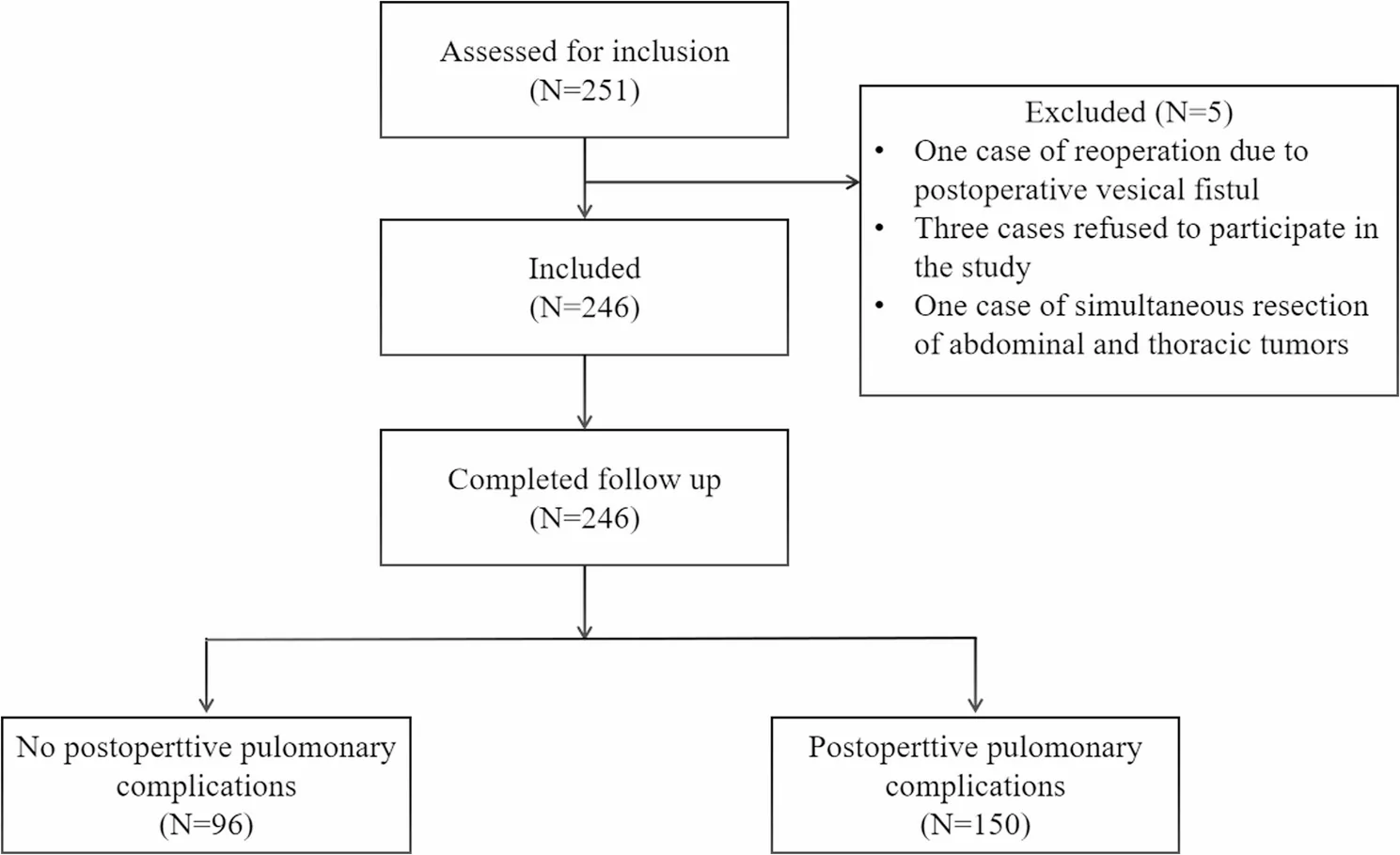
Flow diagram of patient enrollment and follow-up

In this study, a total of 251 patients were screened. Based on the exclusion criteria, 5 patients were eventually excluded: 1 case of reoperation due to postoperative bladder fistula, 3 cases refused to participate in the study, 1 case of simultaneous resection of abdominal and thoracic tumors (Fig. 3). Finally, 246 children were included in the analysis. Baseline characteristics of the study population, stratified by the presence or absence of PPCs, are summarized in Table 1.

**Table 1:** Baseline characteristics of the study population according to the occurrence of PPCs

Variables	Total (*n* = 246)	No PPCs (*n* = 96)	PPCs (*n* = 150)	*P*
Age, y, M (Q₁, Q₃)	4.00 (2.00, 6.00)	5.00 (2.00, 9.00)	3.00 (2.00, 5.00)	**< 0.001**
Sex, *n* (%)				0.065
female	128 (52.03)	57 (59.38)	71 (47.33)	
male	118 (47.97)	39 (40.62)	79 (52.67)	
BMI, kg/m², M (Q₁, Q₃)	15.36 (14.10, 16.43)	15.46 (14.44, 16.89)	15.30 (14.01, 16.02)	0.079
ASA, *n* (%)				**< 0.001**
II	85 (34.55)	59 (61.46)	26 (17.33)	
III	161 (65.45)	37 (38.54)	124 (82.67)	
Recent upper respiratory tract infection, *n* (%)				**< 0.001**
no	178 (72.36)	89 (92.71)	89 (59.33)	
yes	68 (27.64)	7 (7.29)	61 (40.67)	
WBC, ×10⁹/L, M (Q₁, Q₃)	4.93 (3.42, 7.38)	5.02 (3.42, 6.92)	4.90 (3.46, 8.08)	0.275
NE, ×10⁹/L, M (Q₁, Q₃)	2.48 (1.65, 3.89)	2.39 (1.54, 3.66)	2.53 (1.69, 4.25)	0.143
NE%, M (Q₁, Q₃)	0.56 (0.44, 0.67)	0.54 (0.42, 0.65)	0.57 (0.45, 0.69)	0.135
HB, g/L, M (Q₁, Q₃)	102.00 (91.00, 114.00)	103.00 (93.50, 116.00)	102.00 (89.25, 113.00)	0.280
PCT, ng/mL, M (Q₁, Q₃)	0.08 (0.05, 0.15)	0.09 (0.04, 0.18)	0.08 (0.05, 0.12)	0.839
CRP, mg/L, M (Q₁, Q₃)	1.79 (0.41, 5.21)	1.85 (0.40, 5.86)	1.71 (0.51, 4.83)	0.711
NT-proBNP, pg/mL, M (Q₁, Q₃)	80.60 (44.27, 145.50)	81.07 (49.05, 188.50)	78.90 (43.28, 128.75)	0.387
Albumin, g/L, M (Q₁, Q₃)	43.85 (42.00, 45.77)	43.45 (41.38, 45.42)	44.20 (42.32, 46.00)	0.061
PA, g/L, M (Q₁, Q₃)	0.19 (0.16, 0.22)	0.20 (0.16, 0.23)	0.18 (0.16, 0.21)	0.087
SCr, µmol/L, M (Q₁, Q₃)	27.10 (21.72, 35.55)	30.90 (24.73, 40.57)	25.40 (20.35, 32.40)	**< 0.001**
BUN, mmol/L, M (Q₁, Q₃)	4.40 (3.42, 5.27)	4.50 (3.58, 5.60)	4.20 (3.32, 5.18)	0.148
Anesthesiologist Hierarchy, *n* (%)				0.969
Resident	108 (43.90)	42 (43.75)	66 (44.00)	
Attending	138 (56.10)	54 (56.25)	84 (56.00)	
Diagnosis, *n* (%)				**< 0.001**
Neuroblastic tumor	84 (34.15)	17 (17.71)	67 (44.67)	
Hepatoblastoma	61 (24.80)	13 (13.54)	48 (32.00)	
Wilms tumor	35 (14.23)	18 (18.75)	17 (11.33)	
Pelvic rhabdomyosarcoma	46 (18.70)	34 (35.42)	12 (8.00)	
Others	20 (8.13)	14 (14.58)	6 (4.00)	
Tumor Length, cm, M (Q₁, Q₃)	4.40 (2.40, 7.00)	3.45 (2.18, 5.95)	5.00 (2.73, 7.20)	**0.002**
Tumor Width, cm, M (Q₁, Q₃)	3.20 (1.80, 5.50)	2.60 (1.60, 4.65)	3.85 (2.30, 5.70)	**0.004**
Tumor Height, cm, M (Q₁, Q₃)	2.90 (1.60, 4.68)	2.20 (1.17, 3.90)	3.35 (1.70, 5.10)	**0.003**
Tumor Volume, cm³, M (Q₁, Q₃)	20.44 (4.01, 97.20)	10.18 (1.82, 60.04)	38.92 (5.29, 103.72)	**0.003**
Preoperative chemotherapy, *n* (%)				**0.015**
no	39 (15.85)	22 (22.92)	17 (11.33)	
yes	207 (84.15)	74 (77.08)	133 (88.67)	
Reoperation or multiple operations, *n* (%)				**0.003**
no	149 (60.57)	47 (48.96)	102 (68.00)	
yes	97 (39.43)	49 (51.04)	48 (32.00)	
Preoperative blood transfusion, *n* (%)				**0.031**
no	217 (88.21)	90 (93.75)	127 (84.67)	
yes	29 (11.79)	6 (6.25)	23 (15.33)	
Preoperative hypoalbuminemia, *n* (%)				0.517
no	224 (91.06)	86 (89.58)	138 (92.00)	
yes	22 (8.94)	10 (10.42)	12 (8.00)	

*P* values in bold indicate < 0.05

*Abbreviations*: *BMI* Body Mass Index, *ASA* American Society of Anesthesiologists, *WBC* White Blood Cell, *NE* Neutrophil Count, *NE%* Neutrophil Percentage, *HB* Hemoglobin, *PCT* Procalcitonin, *CRP* C-reactive Protein, *NT-proBNP* N-terminal pro-B-type natriuretic peptide, *PA* Prealbumin, *SCr* Serum Creatinine, *BUN* Blood Urea Nitrogen

Z: Mann-Whitney test, χ²: Chi-square test

M: Median, Q_1_: 1st Quartile, Q_3_: 3st Quartile

### Incidence of PPCs

**Fig. 4:**
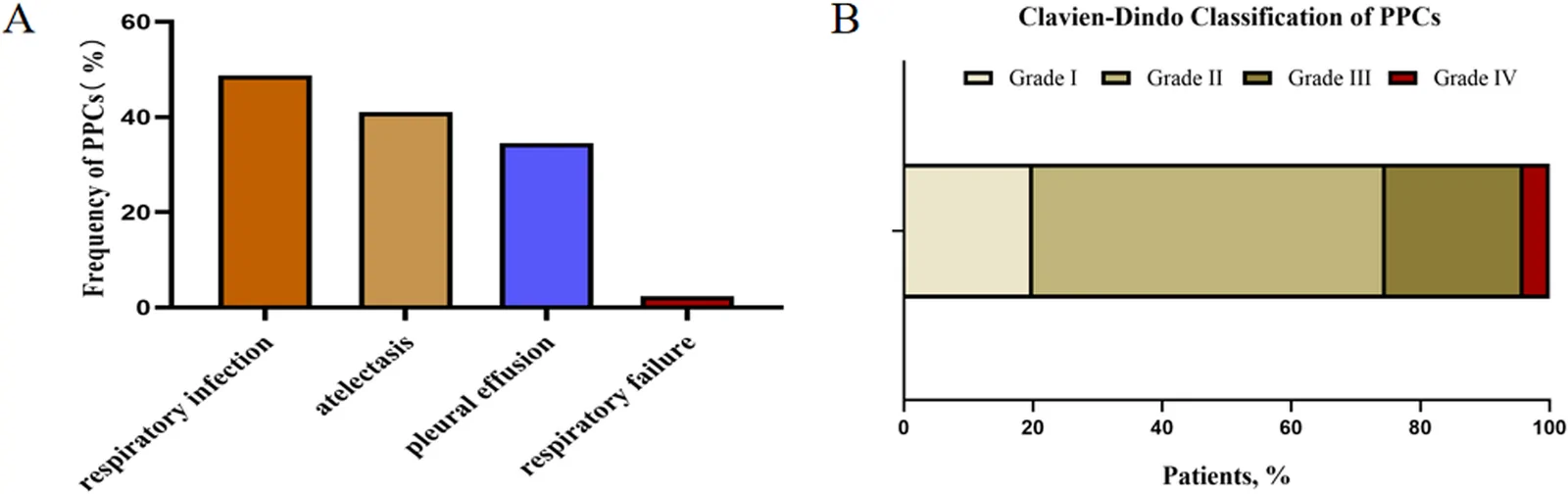
Distribution, incidence, and the Clavien-Dindo Classification of postoperative pulmonary complications. **A** The frequency and proportion of each subtype of PPCs in pediatric patients undergoing abdominal malignant tumor resection. **B** The distribution of grades according to the Clavien-Dindo Classification

The overall incidence of PPCs was 60.98% (150/246). Among the 150 patients who developed PPCs, the complications listed in descending order of frequency were respiratory infection (48.78%, *n* = 120), atelectasis (41.06%, *n* = 101), pleural effusion (34.55%, *n* = 85), and respiratory failure (2.44%, *n* = 6) (Fig. 4). In total, 80.0% (120/150) presented with multiple pulmonary complications in the PPCs group (i.e., two or more concurrent complications).

The distribution of complications according to the Clavien-Dindo Classification was as follows: Grade I, (20%, *n* = 30); Grade II, (54.67%, *n* = 82); Grade III, (21.33%, *n* = 32 ); Grade IV, (4.0%, *n* = 6) ; and no Grade V in-hospital deaths occurred (Fig. 4).

### Comparison of intraoperative and postoperative parameters between the two groups

Compared with the No PPCs group, the PPCs group showed significant differences in the multiple perioperative indicators (all *P* < 0.05), including duration of mechanical ventilation, duration of surgery, intraoperative urine output, peak airway pressure (PIP), plateau airway pressure (Pplat), dynamic lung compliance (Cdyc), proportion of intraoperative blood transfusion, proportion of intraoperative albumin transfusion, and postoperative hospital stay (Table 2).

**Table 2:** Intraoperative and postoperative clinical data according to the occurrence of PPCs

Variables	Total (*n* = 246)	No PPCs (*n* = 96)	PPCs (*n* = 150)	*P*
Duration of mechanical ventilation, min, M (Q₁, Q₃)	210.00 (160.00, 273.75)	175.00 (130.00, 230.00)	230.00 (180.00, 307.50)	**< 0.001**
Duration of surgery, min, M (Q₁, Q₃)	170.00 (121.25, 226.75)	135.00 (104.25, 193.50)	193.50 (140.00, 260.00)	**< 0.001**
Duration of PACU Stay, min, M (Q₁, Q₃)	25.00 (15.00, 30.00)	20.00 (15.00, 30.00)	25.00 (15.00, 30.00)	0.896
Intraoperative Fluid Volume ml·kg⁻¹·h⁻¹, M (Q₁, Q₃)	11.61 (8.55, 15.10)	12.42 (8.63, 16.10)	10.94 (8.53, 14.29)	0.112
Intraoperative Blood Loss, ml, M (Q₁, Q₃)	40.00 (20.00, 50.00)	20.00 (10.00, 50.00)	50.00 (20.00, 50.00)	**< 0.001**
Intraoperative Urine Output, mL·kg⁻¹·h⁻¹, M (Q₁, Q₃)	1.85 (1.24, 2.63)	2.14 (1.37, 2.87)	1.65 (1.20, 2.49)	**0.012**
PIP, cm H₂O, M (Q₁, Q₃)	15.00 (13.00, 18.00)	15.00 (13.00, 16.00)	16.00 (14.00, 18.00)	**< 0.001**
Pplat, cm H₂O, M (Q₁, Q₃)	14.00 (12.00, 15.00)	13.00 (11.75, 14.25)	14.00 (12.00, 16.00)	**0.006**
DP, cm H₂O, M (Q₁, Q₃)	10.00 (9.00, 12.00)	10.00 (9.00, 12.00)	11.00 (9.00, 13.00)	0.122
Cdyc, ml/cm H₂O, M (Q₁, Q₃)	18.45 (12.80, 25.58)	22.25 (13.62, 33.45)	16.75 (12.65, 23.12)	**0.002**
PEEP, cm H₂O, M (Q₁, Q₃)	3.00 (3.00, 3.00)	3.00 (3.00, 3.00)	3.00 (3.00, 3.00)	0.867
Intraoperative blood transfusion, *n* (%)				**0.028**
no	148 (60.16)	66 (68.75)	82 (54.67)	
yes	98 (39.84)	30 (31.25)	68 (45.33)	
Intraoperative albumin transfusion, *n* (%)				**< 0.001**
no	197 (80.08)	88 (91.67)	109 (72.67)	
yes	49 (19.92)	8 (8.33)	41 (27.33)	
Lung Ultrasound Aeration Score, M (Q₁, Q₃)	10.00 (8.00, 13.00)	7.00 (5.00, 10.00)	12.00 (10.00, 14.00)	**< 0.001**
Lung Ultrasound Aeration Score anterior, M (Q₁, Q₃)	2.00 (0.00, 3.00)	1.00 (0.00, 2.00)	2.00 (1.00, 3.00)	**< 0.001**
Lung Ultrasound Aeration Score lateral, M (Q₁, Q₃)	3.00 (1.00, 4.00)	2.00 (0.00, 3.00)	3.00 (2.00, 4.00)	**< 0.001**
Lung Ultrasound Aeration Score posterior, M (Q₁, Q₃)	6.00 (4.00, 8.00)	5.00 (3.00, 6.00)	7.00 (5.00, 8.00)	**< 0.001**
Lung Ultrasound Score of B lines, M (Q₁, Q₃)	11.00 (8.00, 14.00)	8.00 (6.00, 11.00)	13.00 (10.00, 15.00)	**< 0.001**
Lung Ultrasound Score of consolidations, M (Q₁, Q₃)	1.00 (0.00, 3.00)	0.00 (0.00, 2.00)	2.00 (0.00, 4.00)	**< 0.001**
Lung Ultrasound Score of pleural effusion, M (Q₁, Q₃)	0.00 (0.00, 0.00)	0.00 (0.00, 0.00)	0.00 (0.00, 0.00)	0.056
Length of Postoperative Hospital Stay, d, M (Q₁, Q₃)	8.00 (7.00, 10.00)	6.00 (6.00, 8.00)	9.00 (8.00, 12.00)	**< 0.001**

*P* values in bold indicate < 0.05

*Abbreviations*: *PIP* Peak Inspiratory Pressure, *Pplat* Plateau Pressure, *DP* Driving Pressure, *Cdyc* Dynamic Lung Compliance, *PEEP* Positive end-expiratory pressure

Z: Mann-Whitney test, χ²: Chi-square test

M: Median, Q_1_: 1st Quartile, Q_3_: 3st Quartile

The results demonstrated that the No PPCs group exhibited significantly lower global LUSS and regional aeration scores for the anterior, lateral and posterior chest regions compared with the PPCs group (*P* < 0.001). Similarly, B-line and lung consolidation scores were also markedly lower in the No PPCs group (*P* < 0.001), whereas no significant difference was observed in pleural effusion scores between the two groups (Table 2).

### Univariate and multivariate analyses of risk factors for PPCs

Univariate logistic regression analysis identified fifteen clinical factors associated with the occurrence of PPCs (Table 3). Using the least absolute shrinkage and selection operator (LASSO) regression with the lambda.1se criterion (λ = 0.0596), we identified 5 non-zero coefficient predictors: duration of mechanical ventilation, PACU LUSS, ASA classification, recent URI, and Intraoperative albumin transfusion (Fig. S1). These variables were subsequently included in the final multivariable logistic regression model.

**Table 3:** Univariate and multivariate logistic regression analyses of risk factors for PPCs

Variables	Univariate		Multivariate	
	OR (95%CI)	*P*	OR (95%CI)	*P*
Age	0.86 (0.80 ~ 0.93)	**< 0.001**		
ASA				
II	1.00 (Reference)		1.00 (Reference)	
III	7.60 (4.22 ~ 13.71)	**< 0.001**	3.60 (1.77 ~ 7.34)	**< 0.001**
Recent upper respiratory tract infection				
no	1.00 (Reference)		1.00 (Reference)	
yes	8.71 (3.78 ~ 20.09)	**< 0.001**	4.71 (1.84 ~ 12.08)	**0.001**
Tumor Volume	1.01 (1.01 ~ 1.01)	**0.033**		
Preoperative chemotherapy				
no	1.00 (Reference)			
yes	2.33 (1.16 ~ 4.65)	**0.017**		
Reoperation or multiple operations				
no	1.00 (Reference)			
yes	0.45 (0.27 ~ 0.76)	**0.003**		
Preoperative blood transfusion				
no	1.00 (Reference)			
yes	2.72 (1.06 ~ 6.94)	**0.037**		
Duration of mechanical ventilation	1.01 (1.01 ~ 1.01)	**< 0.001**	1.01 (1.01 ~ 1.01)	**0.002**
PIP	1.17 (1.06 ~ 1.29)	**0.001**		
Pplat	1.15 (1.04 ~ 1.27)	**0.008**		
Cdyc	0.96 (0.94 ~ 0.98)	**< 0.001**		
PEEP				
no	1.00 (Reference)			
yes	0.12 (0.02 ~ 0.95)	**0.044**		
Lung Ultrasound Aeration Score	1.39 (1.27 ~ 1.53)	**< 0.001**	1.29 (1.17 ~ 1.42)	**< 0.001**
Intraoperative blood transfusion				
no	1.00 (Reference)			
yes	1.82 (1.07 ~ 3.12)	**0.029**		
Intraoperative albumin transfusion				
no	1.00 (Reference)			
yes	4.14 (1.84 ~ 9.28)	**< 0.001**		

*P* values in bold indicate < 0.05

*Abbreviations*: *ASA* American Society of Anesthesiologists, *PIP* Peak Inspiratory Pressure, *Pplat* Plateau Pressure, *Cdyc* Dynamic Lung Compliance, *PEEP* Positive end-expiratory pressure, *OR* Odds Ratio, *CI* Confidence Interval

Subsequent multivariate logistic regression analysis further confirmed four independent risk factors for PPCs: ASA classification (OR = 3.60, 95% CI = 1.77–7.34, *P* < 0.001), recent URI (OR = 4.71, 95% CI = 1.84–12.08, *P* = 0.001), duration of mechanical ventilation (OR = 1.01, 95% CI = 1.01–1.01, *P* = 0.002), and PACU LUSS (OR = 1.29, 95% CI = 1.17–1.42, *P* < 0.001) (Table 3).

### Predictive performance of the combined predictive model and the PACU LUSS for PPCs

**Fig. 5:**
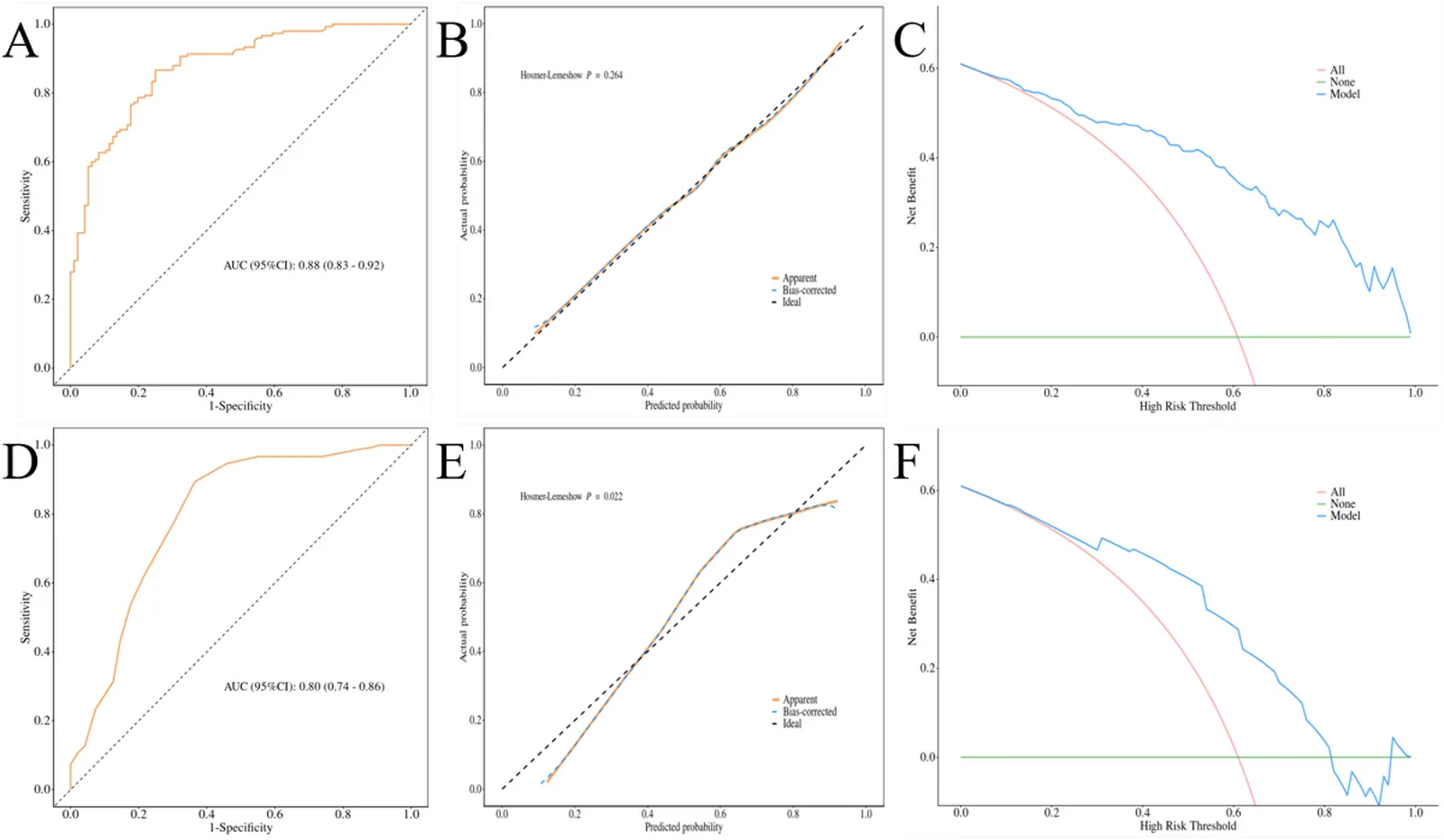
Predictive performance of the combined predictive model and the PACU LUSS for PPCs. **A** ROC curve for the combined prediction model incorporating PACU-LUS score, ASA classification, recent URI, and mechanical ventilation duration. **B** Hosmer-Lemeshow calibration curve for the combined predictive model (*P* = 0.264). **C** Decision curve analysis (DCA) for the combined predictive model. **D** ROC curve for the standalone PACU-LUS aeration score. **E** Hosmer-Lemeshow calibration curve for the standalone PACU-LUS score (*P* = 0.022). **F** Decision curve analysis (DCA) for the standalone PACU-LUS aeration score

ROC curve analysis was conducted to evaluate the predictive value of PACU LUSS alone and in combination with the other three independent risk factors for PPCs. The analysis revealed that the PACU LUSS had a predictive AUC of 0.80 (sensitivity = 0.89, specificity = 0.64) for PPCs, the optimal cut-off value of the LUSS was 8.5. A combined predictive model integrating the PACU LUSS, ASA classification, recent URI, and duration of mechanical ventilation showed superior predictive efficiency, with an AUC of 0.88, sensitivity of 0.87 and specificity of 0.75 (Fig. 5). These findings indicate that PACU LUSS may have important clinical predictive value for the occurrence of PPCs.

DCA results demonstrated that the combined model generated substantially higher net clinical benefit than the “treat all” and “treat none” strategies across most high-risk threshold probability ranges. By contrast, the standalone PACU LUSS curve presented a narrower range of positive net clinical benefit and converged with the “treat none” curve at high threshold probabilities, indicating weaker clinical utility relative to the four-variable combined model (Fig. 5).

### Age-stratified subgroup analysis

Patients were stratified by an age threshold of 2 years based on developmental differences in pediatric respiratory physiology. The PPCs incidence was 57.76% (93/161) in children aged > 2 years and 67.06% (57/85) in children aged ≤ 2 years.

In the subgroup aged > 2 years, multivariate logistic regression analysis identified four independent risk factors for PPCs: ASA classification (OR = 3.17, 95% CI = 1.32–7.59, *P* = 0.010), recent URI (OR = 14.58, 95% CI = 1.80–118.24, *P* = 0.012), duration of mechanical ventilation (OR = 1.01, 95% CI = 1.01–1.01, *P* = 0.006), and PACU LUSS (OR = 1.23, 95% CI = 1.09–1.39, *P* < 0.001) (Table S1).

In the subgroup aged ≤ 2 years, multivariate logistic regression analysis identified three independent risk factors for PPCs: ASA classification (OR = 6.59, 95% CI = 1.69–25.76, *P* = 0.007), PACU LUSS (OR = 1.44, 95% CI = 1.19–1.75, *P* < 0.001), and intraoperative albumin administration (OR = 9.98, 95% CI = 1.07–93.09, *P* = 0.043) (Table S2).

## Discussion

This single-center prospective observational study demonstrated that the overall incidence of PPCs in pediatric patients undergoing abdominal malignant tumor resection was 60.98%. Multivariate logistic regression identified higher ASA physical status, recent URI, duration of mechanical ventilation, and PACU LUSS as independent risk factors for PPCs. The PACU LUSS alone showed good predictive performance (AUC = 0.80), and the combined model incorporating all four independent risk factors achieved higher predictive accuracy (AUC = 0.88). These findings confirm the high risk of PPCs in children undergoing abdominal tumor surgery and verify that PACU LUSS can be used for early risk assessment, supporting perioperative lung protection in pediatric oncology patients.

The incidence of PPCs in this study was 60.98%, which was significantly higher than that in adults undergoing emergency abdominal surgery (22.5%) [32], non-cardiothoracic surgery (33.4%) [33], as well as in children undergoing major abdominal surgery (26.1%) [34] and otolaryngology surgery(2.2%) [35]. These differences clearly reflect the impact of abdominal malignant tumor surgery on the pediatric respiratory system, which can be explained from two aspects. First, compared with adults, children have narrower airways, lower lung compliance, weaker respiratory compensation, and are more sensitive to anesthesia and surgical stress [11]. Second, compared with other pediatric surgeries, abdominal malignant tumor resection is more invasive, longer in operation time, and causes more significant interference to the diaphragm and thoracic cavity [36]. In addition, children with tumors often suffer from malnutrition and poor baseline conditions, further increasing the risk of complications. Notably, 80% of the children in this study had multiple complications. The superposition of multiple pathological factors leads to a high incidence and complexity of complications, which not only delays recovery and increases medical expenses, but may also cause acute respiratory insufficiency and increase the family burden.

Multivariate analysis confirmed that ASA classification was an independent risk factor (OR = 3.60), which was consistent with adult studies [8, 37] and indicated that baseline physiological status had a more significant impact on children. Children with higher ASA grades often have decreased cardiopulmonary reserve, malnutrition or tumor-related systemic diseases, which aggravate perioperative stress and increase susceptibility to pulmonary complications. These results emphasize the importance of preoperative optimization, including nutritional support, infection control and cardiopulmonary function adjustment, especially for children with higher ASA grades.

Recent URI was another independent predictor (OR = 4.71). Preoperative upper respiratory tract inflammation increases airway hyperresponsiveness and secretion production, which are further aggravated by tracheal intubation and mechanical ventilation [38],and pediatric patients with preoperative URI are prone to perioperative respiratory adverse events [39].Postoperative ciliary clearance dysfunction further increases the risk of atelectasis and lower respiratory tract infection. These findings suggest that comprehensive preoperative preparation should be performed for children with recent URI, and elective surgery may be postponed when necessary. Recent URI (within 4 weeks preoperatively) was identified as an independent risk factor for postoperative pulmonary complications in children undergoing abdominal tumor resection. Accordingly, optimal surgical timing requires careful balancing between respiratory complication risk and tumor progression. For indolent tumors, postponing surgery for ≥ 2 weeks after complete URI symptom resolution is advisable. In contrast, for aggressive malignancies, surgery may proceed under optimized respiratory management with concurrent preoperative chemotherapy and dynamic tumor marker monitoring. For tumor rupture or unequivocal progression, emergency surgery is warranted despite elevated respiratory risk. Across all scenarios, decisions should be individualized through multidisciplinary team discussions. Further prospective studies are warranted to establish evidence-based guidelines for this vulnerable population.

Prolonged duration of mechanical ventilation was independently associated with PPCs (OR = 1.01). Prolonged positive-pressure ventilation contributes to ventilator-induced lung injury [40], alveolar collapse, and impaired diaphragmatic function, even with protective ventilation strategies [40–42]. Shorter anesthesia and ventilation time, along with early extubation and spontaneous breathing rehabilitation, may help reduce pulmonary complications in this population.

The most clinically significant finding of this study is that PACU LUSS is a strong independent risk factor for PPCs (OR = 1.29) with excellent predictive value (AUC = 0.80, sensitivity 0.89, specificity 0.64). These results extend the evidence of LUS prediction from adults [17, 18] to children undergoing abdominal tumor surgery. Compared with chest X-ray and CT, LUS is non-invasive, radiation-free, bedside applicable, and highly sensitive to early pulmonary ventilation loss, B-line formation and consolidation [16, 43, 44]. The 12-region pediatric LUS scoring system used in this study can comprehensively reflect the global and regional ventilation impairment immediately after surgery [31]. The model constructed by combining this score with ASA classification, recent URI and duration of mechanical ventilation achieved an AUC of 0.88 with improved specificity, supporting its use in routine perioperative risk stratification.

Subgroup analysis revealed marked differences in risk factor profiles between children aged ≤ 2 years and those aged > 2 years, which can be attributed to immature alveolar development and insufficient endogenous albumin reserves in infants and toddlers. The four core risk factors identified in the overall cohort remained independent predictors of PPCs among children older than 2 years. In contrast, intraoperative albumin transfusion emerged as an independent risk factor exclusively in the subgroup of children aged ≤ 2 years (OR = 9.98). Infants possess an underdeveloped alveolar-capillary barrier; excessive exogenous albumin infusion may exacerbate pulmonary interstitial edema and further increase the risk of PPCs. Clinically, these findings indicate that physicians should rigorously restrict unnecessary intraoperative albumin supplementation for young children.

Based on the independent risk factors identified by multivariate regression analysis, we recommend a tiered perioperative lung-protective strategy for routine clinical practice. Preoperatively, nutritional and cardiopulmonary reserve should be optimized in children with high ASA status, and elective surgery should be delayed for those with unresolved upper respiratory tract infection until symptoms and inflammatory markers have normalized for at least two weeks. Intraoperatively, mechanical ventilation duration should be minimized, individualized lung-protective ventilation with precise PEEP titration should be applied, and excessive albumin infusion should be strictly avoided in children aged ≤ 2 years. In the PACU, standardized bedside lung ultrasound can be performed promptly after extubation to determine LUSS; intensive respiratory monitoring and chest physiotherapy are recommended for patients with LUSS above the optimal cutoff of 8.5.

This study has several limitations. First, this was a single-center observational study conducted at a tertiary cancer hospital, which may have introduced selection bias. Moreover, differences in perioperative management strategies and baseline patient characteristics across healthcare settings of varying levels warrant caution in generalizing the present findings.Second, the follow-up period was limited to one week postoperatively. Given that delayed PPCs may occur beyond seven days after surgery or after hospital discharge, the current follow-up protocol may have underestimated the true incidence of PPCs and precluded assessment of long-term pulmonary outcomes. Third, during LUS examinations, children often cry, move, and fail to cooperate, making it difficult to maintain a fixed position, which may result in image artifacts and scoring bias that cannot be entirely eliminated. Fourth, routine preoperative LUS was not performed in this study, and data on the patients’ preoperative baseline ventilation status were lacking. Consequently, pre-existing atelectasis could not be reliably distinguished from newly developed postoperative pulmonary lesions, which, to some extent, limits causal inference regarding the relationship between surgical factors and the development of PPCs. Fifth, postoperative pain scores were not systematically collected, nor was postoperative pain intensity incorporated into the multivariable regression model as a potential risk factor. Postoperative pain can lead to shallow and rapid breathing and impaired cough and sputum clearance, representing an important trigger for PPCs. The omission of this variable may have introduced residual confounding and compromised the comprehensiveness of risk factor identification in the model. Sixth, the sample size in some subgroup analyses was relatively limited, which may have resulted in insufficient statistical power and thereby underestimated the true associations between certain factors and PPCs. In summary, future multicenter, large-scale prospective studies incorporating preoperative LUS baseline assessment, systematic postoperative pain monitoring, and longer postoperative follow-up are warranted to further validate the present findings and optimize perioperative lung protection strategies for children undergoing abdominal tumor surgery.

## Conclusion

In conclusion, within the present cohort, children undergoing abdominal malignant tumor resection exhibited a relatively high incidence of PPCs. Higher ASA classification, recent URI, prolonged mechanical ventilation, and PACU LUSS may be associated with an increased risk of PPCs. As a noninvasive bedside assessment tool, PACU LUSS may provide reference information for PPCs risk evaluation; when integrated with clinical risk factors, it may facilitate preliminary risk stratification by clinicians. Routine application of PACU LUSS in clinical practice may facilitate the early identification of children at elevated risk, thereby providing a reference for optimizing perioperative management in this vulnerable population. Given the limitations of the present observational study, these conclusions warrant further validation in multicenter prospective studies to assess their external generalizability.

## Supplementary Information

Supplementary Material 1.

## Data Availability

Data will be available from the corresponding author when required.

## Abbreviations

PPCs: Postoperative pulmonary complications
PACU: Post-anesthesia care unit
LUSS: Lung ultrasound aeration score
ROC: Receiver operating characteristic
DCA: Decision curve analysis
ASA: American Society of Anesthesiologists
URI: Recent upper respiratory tract infection
AUC: Area Under the ROC Curve
LUS: Lung ultrasound
CT: Computed Tomography
ETCO_2_: End-tidal carbon dioxide
PEEP: Positive end-expiratory pressure
BMI: Body mass index
NT-proBNP: N-terminal pro-B-type natriuretic peptide
EPV: Events per variable
PIP: Peak Inspiratory Pressure
Pplat: Plateau Pressure
DP: Driving Pressure
Cdyc: Dynamic Lung Compliance
