# Supplementary material for: Risk factors and predictive value of lung ultrasound aeration score for postoperative pulmonary complications in pediatric patients undergoing abdominal tumor resection: a prospective observational study
Source: BMC Anesthesiol. 2026 Sep 19;26:659. doi: 10.1186/s12871-026-04265-1 (PMC13737555; doi:10.1186/s12871-026-04265-1)
Supplement: Supplementary file 1 [file 12871_2026_4265_MOESM1_ESM.docx]

**Supplementary Table S1. multivariate logistic regression analyses of risk factors for PPCs in the subgroup of children aged > 2 years**

| **Variables** | **Multivariate** | |
| --- | --- | --- |
|  | OR (95%CI) | P |
| ASA |  |  |
| II | 1.00 (Reference) |  |
| III | 3.17 (1.32 ~ 7.59) | **0.010** |
| Recent upper respiratory  tract infection |  |  |
| no | 1.00 (Reference) |  |
| yes | 14.58 (1.80 ~ 118.24) | **0.012** |
| Duration of mechanical ventilation | 1.01 (1.01 ~ 1.01) | **0.006** |
| Lung Ultrasound Aeration Score | 1.23 (1.09 ~ 1.39) | **<0.001** |

OR: Odds Ratio, CI: Confidence Interval

Abbreviations: ASA, American Society of Anesthesiologists.

**Supplementary Table S2. multivariate logistic regression analyses of risk factors for PPCs in the subgroup of children aged ≤ 2 years**

| **Variables** | **Multivariate** | |
| --- | --- | --- |
|  | OR (95%CI) | P |
| ASA |  |  |
| II | 1.00 (Reference) |  |
| III | 6.59 (1.69 ~ 25.76) | **0.007** |
| Lung Ultrasound Aeration Score | 1.44 (1.19 ~ 1.75) | **<0.001** |
| Intraoperative albumin transfusion |  |  |
| no | 1.00 (Reference) |  |
| yes | 9.98 (1.07 ~ 93.09) | **0.043** |

OR: Odds Ratio, CI: Confidence Interval

Abbreviations: ASA, American Society of Anesthesiologists.

**Supplementary Fig S1.Variable selection using LASSO regression.**

(A) LASSO coefficient profiles of all candidate predictors. Each colored line represents one variable. The vertical dashed line indicates the optimal penalty parameter. (B) Ten-fold cross-validation of LASSO regression. The red line shows the mean binomial deviance, and the gray area represents the standard error. The left and right vertical dashed lines denote λ.min (minimum deviance) and λ.1se (one standard error from the minimum), respectively. (C) Boxplots of selected variables under λ.min (left) and λ.1se (right) schemes.
